# Bisphenols induce human genomic damage and modulate HERVs/*env* expression

**DOI:** 10.1002/em.22499

**Published:** 2022-09-01

**Authors:** Stefano Ruberto, Alfredo Santovito, Elena R. Simula, Marta Noli, Maria A. Manca, Leonardo A. Sechi

**Affiliations:** ^1^ Department of Biomedical Sciences Division of Microbiology and Virology, University of Sassari Sassari Italy; ^2^ Department of Life Sciences and Systems Biology University of Turin Torino Italy

**Keywords:** BPA, BPF, BPS, genotoxicology, HERVs, micronucleus

## Abstract

Bisphenol A (BPA), a recognized endocrine‐disrupting chemical, is used in the production of epoxy and polycarbonate resins. Since human exposure to BPA has been associated with increased cancer susceptibility, the market has shifted to products often labeled as “*BPA free*” containing BPA analogs such as bisphenol F (BPF) and bisphenol S (BPS). However, the European legislation on BPF and BPS is still unclear. This study analyzed the effects of BPA, BPF, and BPS exposure on human peripheral blood mononuclear cells by using *in vitro* micronucleus assay. Furthermore, it investigated the impact of bisphenols exposure on human endogenous retroviruses (HERVs) expression, which is implicated with the pathogenesis of several human diseases. The micronucleus assay revealed a significant genotoxic effect in peripheral blood cells after exposure to BPA and BPF at concentrations of 0.1, 0.05, and 0.025 μg/ml, and to BPS at 0.1 and 0.05 μg/ml. In addition, BPA exposure seems to upregulate the expression of HERVs, while a downregulation was observed after BPF and BPS treatments. Overall, our data showed the toxic effect of BPA and its analogs on circulating cells in the blood and demonstrated that they could modulate the HERVs expression.

**Abbreviations**
BNC: binucleated cell
BPA: bisphenol A
BPF: bisphenol F
BPS: bisphenol S
BP: bisphenol
CA: chromosomal aberration
CBPI: cytokinesis‐block proliferation index
DMSO: dimethyl sulfoxide
ECHA: European Chemical Agency
EDC: Endocrine‐disrupting compound
EFSA: European Food Safety Authority
ERV: Endogenous retrovirus
FCM: food contact material
HERV: human endogenous retrovirus
MCF‐7: Michigan Cancer Foundation‐7
MI: Mitotic index
MMC: mitomycin‐C
MNC: micronucleated cell
MNi: micronuclei
NBUD: nuclear bud
NPB: nucleoplasmic bridge
RD: reference dose
RNS: reactive nitrogen species
ROS: reactive oxygen species
SINE: short interspersed elements
SML: specific migration limit
STING: stimulator of interferon gene
US EPA: US Environmental Protection Agency

## 1 INTRODUCTION

Bisphenols are commonly used in various industrial and commercial applications. These compounds are estrogen‐like and can be found in several environments (Huang et al., 2012). One of these is bisphenol A (BPA; 4,4′‐propane‐2,2‐diyldiphenol, CAS no. 80‐05‐7), which is used in the production of various food container materials, glass jars, bottles, and metal lids (Bailin et al., 2008; Huang et al., 2012; Liao et al., 2012a). Concerns have been raised about the possible effects of BPA on human health. It has been known that it can affect the hormonal activity of the human body and cause cancer and obesity (Ariemma et al., 2016; Toft et al., 2004). Due to its estrogen‐like effect, it has been recognized as endocrine‐disrupting chemicals (EDCs) with carcinogenic and genotoxic effects (Doherty et al., 2010). The European Chemical Agency (ECHA) placed BPA on list of substances that pose a high concern (ECHA, 2018). In 2018, the EU Commission set stricter regulations regarding the use of this compound, imposing a reference dose (RD) for oral exposure of 0.05 mg/kg body weight (BW)/day (EU, 2018). Due to the strict regulations regarding the use of BPA, structurally similar compounds were introduced, such as bisphenol S (BPS; 4,4′‐Sulfonyldiphenol, CAS no. 80‐09‐1) and bisphenol F (BPF; 4,4′‐Methylenediphenol, CAS no. 620‐92‐8). These BPA analogs are applied in several products marketed as “*BPA free*” given the BPA legal restriction (Liao et al., 2012b). Due to its anticorrosive properties, BPS is used in several applications, such as food and beverage cans, thermal paper, and cosmetics (ANSES, 2017; Pivnenko et al., 2015; Viñas et al., 2010). As for BPF, this latter replaces BPA in plastic, epoxy resins (Goodson et al., 2002), and dental materials such as tissue substitutes and prosthetic devices (Rochester & Bolden, 2015). Several studies reported hormonal effects of BPS and BPF (Eladak et al., 2015) and this is not surprising given their BPA structural analogies. Literature data indicate that exposure to BPA, BPS, and BPF *in vitro* shows cytotoxic effects and DNA damage in terms of increased frequency of chromosomal aberrations (CAs) (Fic et al., 2013; Rochester & Bolden, 2015; Santovito et al., 2018). To date, there is very limited knowledge on the metabolism and genomic effects of BPS and BPF. On the one hand, the use of BPS as a monomer in food contact plastic (FCMs) is limited with a specific migration limit (SML) of 0.05 mg/kg (EFSA et al., 2020). On the other hand, no threshold has been imposed for BPF RD, as its metabolism and biological effects have not been well studied (Munn & Goumenou, 2013).

The first objective of this study is to investigate the effects of exposure to BPA and related compounds on the genome of human peripheral blood cultures. The genomic damage was evaluated by micronuclei (MNi) assay, a fast and inexpensive test able to detect both clastogenic and aneugenic properties of a single chemical or a mixture of different compounds (Santovito et al., 2019). This assay also allows the evaluation of the frequency of nuclear buds (NBUDs), which represent the elimination process of amplified DNA or excess chromosomes from aneuploidy cells (Fenech et al., 2011). Finally, the cytokinesis‐block proliferation index (CBPI) is routinely used to determine the cytotoxicity of a given compound. Cytostasis may result from effects on cell division and may also be involved in cell death (Lorge et al., 2008). Micronucleation can also trigger a cascade of genetic instability, which potentially leads to RNA fragments expression (MacDonald et al., 2020), as endogenous retroviruses (ERVs) (Canadas et al., 2018; Lee et al., 2012) and short interspersed elements (SINEs) (Rudin & Thompson, 2001). Endogenous retroviral sequences were integrated through repeated infections during evolution (Frank & Feschotte, 2017). The 8% of the human genome is composed of genetic elements that have been acquired over the last 100 million years through multiple integrations by exogenous retroviruses, that is, human endogenous retroviruses (HERVs) (Vargiu et al., 2016). Although human retroviruses are not infectious, they can also contribute to the development of the inflammatory condition (Grandi & Tramontano, 2018). Transcriptional activation of HERVs is a common feature in neurodegenerative diseases and human cancers (Giménez‐Orenga & Oltra, 2021; Mao et al., 2021). These may act as cofactors or causative elements that contribute to disease progression and onset (Matteucci et al., 2018). In addition, net loss of heterochromatin with aging may lead to abnormal activation of these retrotransposons (Pal & Tyler, 2016). Several factors as the accumulation of deleterious mutations, deletions, and epigenetic modifications, such as DNA methylation or heterochromatin silencing, can affect the HERVs transcription levels (Hurst & Magiorkinis, 2017). Therefore, MNi can initiate pro‐inflammatory signaling cascades which seem to involve inflammatory and HERVs gene activation (MacDonald et al., 2020). HERVs family expression can be modulated by environmental factors, such as physical agents, external chemical substances, and viral infections (Zhang et al., 2019). Several studies reported the influence on HERV‐H/*env* expression by chemical elements exposure as hydroquinone, cupric ion, and copper sulfate in several tumor cell lines (Conti et al., 2016; Karimi et al., 2019). Wentzensen et al. (2007) and Yi et al. (2006) also reported a HERV‐H RNA sequence overexpressing in several human cancer cell lines and primary tumor tissues, which were correlated to demethylation of HERV‐H. According to Durnaoglu et al. (2021), HERV‐R expression can be down‐ or up‐regulated at sites of inflammation in human vessels and play a role in inflammatory vascular diseases. Furthermore, its differential expression in normal and pathological tissues under physical agents of genomic instability such as γ radiation has been detected (Lee et al., 2012).

On the bases of these assumptions, the second aim of this study is to evaluate the *env* genes transcript levels in HERV‐H and HERV‐R in peripheral blood cultures exposed to several concentrations of BPs and determine the relationship between MNi frequency and the *env* gene expression.

## 2 MATERIALS AND METHODS

### 2.1 Study population

Peripheral venous blood was collected from four healthy subjects who were non‐smokers, not alcoholics, not under drug or radiological treatment, and had no recent exposure to mutagens. All subjects signed the informed consent. The study was approved by the local University ethics committee and conducted according to the ethical standards of the 2013 Declaration of Helsinki.

### 2.2 Blood cultures

Blood samples were obtained by venepuncture (about 10 ml of blood per subject), collected in heparinized tubes (Terumo Europe), and immediately processed. Heparinized venous blood (0.3 ml) was cultured in 25 cm^2^ flasks containing 6 ml of RPMI‐1640 medium, 2 ml of fetal calf serum, 200 μl of the mitogenic agent Phytohemagglutinin‐L (2.3% v/v), and 100 μl of antibiotics solution (100 IU/ml penicillin and 100 μg/ml streptomycin) (Invitrogen‐Life Technologies). Cultures were prepared for two different assays and incubated sequentially at 37°C and under 5% of CO_2_ in the air in a humidified atmosphere. The total time of lymphocyte cultures was 72 h. For the three stock solution preparations, 200 mg of BPA, BPS, and BPF (Sigma‐Aldrich) were first dissolved in 1 ml of DMSO (Sigma‐Aldrich) solution and stored at 4°C until used for the final exposure solutions in a culture medium. After 24 h of incubation, we added BPs in separate cultures at final concentrations of 0.0125, 0.025, 0.05, and 0.1 μg/ml for each one. These concentrations are multiples and submultiples of RD values (0.05 μg/ml) to detect a possible dose‐dependent response in the genomic damage assay. Three control cultures were assessed: (1) solvent control, by addition of only 0.1% of DMSO; (2) positive control, by addition of only 0.1 μg/ml mitomycin‐C (MMC) (Sigma‐Aldrich); and (3) negative control (NC) culture without BPs and DMSO.

### 2.3 Cytokinesis‐block micronucleus assays

The total time of lymphocyte cultures was 72 h, and at the 44th, the cytochalasin‐B (Sigma‐Aldrich) was added to the first set of cultures at a final concentration of 6 μg/ml, to block cytokinesis. After 72 h of incubation at 37°C, the cells were collected by centrifugation (10 min at 640*g*) and treated with a prewarmed hypotonic solution (75 mM KCl; Merck S.p.A.) for 10 min. After centrifugation and removal of the supernatant, the cells were fixed with a solution of methanol/acetic acid (3:1 v/v). The treatment with the fixative was repeated three times. Finally, the supernatant was discarded, and the pellet, dissolved in a minimal volume of fixative, was seeded on the slides to detect MNi by conventional staining with 5% Giemsa (pH 6.8) (Carlo Erba Reagenti) prepared in Sörensen buffer (Merck S.p.A.). Microscope analysis was performed at 1000× magnification on a light microscope (Dialux 20). We evaluated the frequency of MNi and NBUDs in 1000 binucleated lymphocytes with well‐preserved cytoplasm per subject. Cells containing one or more MNi were scored as “micronucleated cells” (MNCs). A total of 1000 lymphocytes per donor per concentration were also scored to evaluate the CBPI, according to the following formula: [1 × *N*1] + [2 × *N*2] + [3 × (*N*3 + *N*4)]/*N*, where *N*1–*N*4 represents the number of cells with 1–4 nuclei, respectively, and *N* is the total number of cells scored.

### 2.4 Sample preparation for gene expression

The second set of cultures was processed by Ficoll‐Histopaque gradient centrifugation (Sigma‐Aldrich) after incubation for 72 h to isolate  PBMCs. Total RNA extraction from PBMCs was performed using the RNeasy Plus Mini kit (Qiagen GmbH) and concentration was assessed using Nanodrop One (Microvolume UV–Vis Spectrophotometer, Thermo Fisher Scientific). For cDNA synthesis, 2 μg of pure mRNA was reverse transcribed in 20 μl final reaction volume using the High‐Capacity cDNA RT kit (Applied Biosystems, Life Technologies) with oligo (dT) primers (Invitrogen, Life Technologies) extra addition to enhancing the RT reaction.

### 2.5 Real‐time PCR

The HERV‐H/*env* and HERV‐R/*env* genes expression in PBMCs from blood exposed to BPs was quantitatively assessed by real‐time PCR. The HERV‐H/*env* primers (Gene Bank Accession no. AJ289711.1; forward primer 5′‐CCCATATTTGGACCTCTCAC‐3′; reverse primer 5′‐TGTGTAGTTGGGCTTTGGAG‐3′) and the HERV‐R/*env* primers (Gene Bank Accession no. NM_001007253.4; forward primer 5′‐GCACGAGTCAGCGGTGAAGA‐3′; reverse primer 5′‐GGGCTCAGGCAATTTCTGGT‐3′) were designed using PerlPrimer software (version 1.1.21) (Marshall, 2004). Primer's amplification efficiency was determined by the slopes of the standard curves obtained by serial dilution. The glyceraldehyde‐3‐phosphate dehydrogenase (GAPDH) gene (Gene Bank Accession no. NM_002046; forward primer 5′‐CAAGGAGTAAGACCCCTGGAC‐3′; reverse primer 5′‐TCTACATGGCAACTGTGAGGAG‐3′) was used as a house‐keeping gene to normalize the results. Real‐time PCR was performed using SYBR Green PCR Master Mix (Applied Biosystems, Thermo Fisher Scientific) and conducted on CFX Connect Real‐Time PCR (Bio‐Rad). RT‐PCR thermal protocol, according to the SYBR Green PCR Master Mix propriety, was carried out at 50° for 2 min, 95° for 2 min, and 50 cycles of 95°C for 15 s and 60° for 1 min. Melting curve analyzes were performed from 65°C to 95°C. All samples were amplified in triplicate. Relative gene expression of samples was calculated by Ct comparative, according to the delta–delta Ct method (2^–∆∆Ct^).

### 2.6 Statistical analysis

The Shapiro–Wilk test was performed to assess the normality of the data distribution. Comparison of the mean values of the percentages of MNi, MNC, NBUDs, and CBPI between different BPs concentrations and controls was assessed with non‐parametric Mann–Whitney test according to the data distribution. Correlation between different BPs concentrations and biomarkers (MNi, cells with MNi, NBUDs, CBPI, and HERVs/*env* expression) was assessed by regression analysis. Parametric data were analyzed by one‐way ANOVA with post hoc Dunnett's test. ANOVA test was used to compare the transcript levels of HERV‐H/*env* and HERV‐R/*env* expression in PBMCs belonging to blood cultures exposed to different BPA, BPF, and BPS concentrations. The correlation between HERV‐H/*env* and HERV‐R/*env* expression and MNi and NBUGs frequencies, respectively, was analyzed using Pearson and Spearman tests. Statistical calculations were performed using GraphPad Prism 8.2.0 software (GraphPad Software) and the SPSS software package (version 28.0; SPSS, Inc.). The level of statistical significance was set at *p* values of 5% or less.

## 3 RESULTS

### 3.1 MNi and NBUDs in cultures exposed to BPs

**FIGURE 1:**
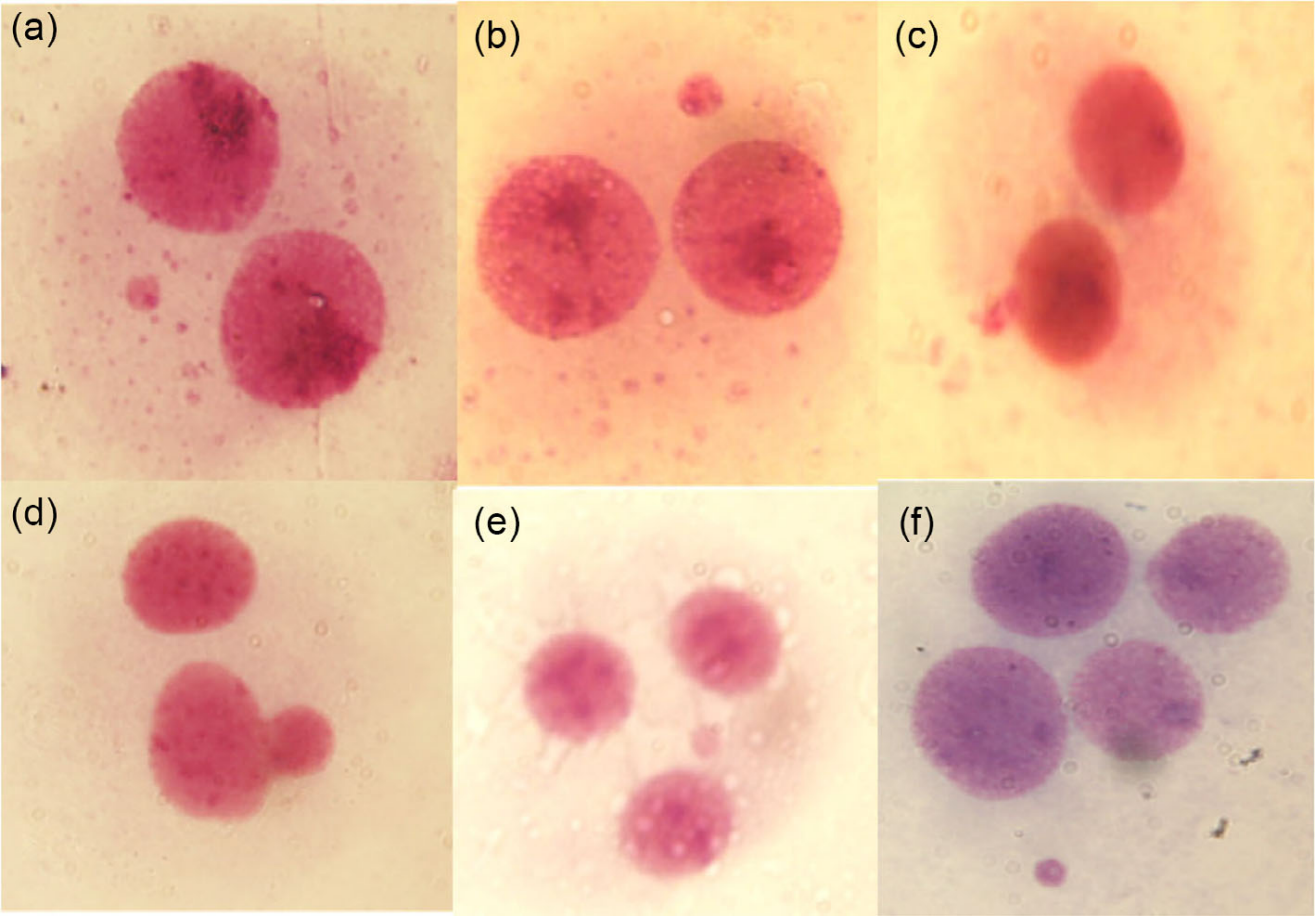
Examples observed bi‐nucleated cells with micronuclei (MNi; a, b), bi‐nucleated cell with nuclear bud (c, d), tri‐nucleated cell with MNi (e), and tetra‐nucleated cell with MNi (f). According to standardized procedures, MNi of tri‐ and tetra‐nucleated cells were not scored in the evaluation of the total genomic damage

In Figure 1, we reported some examples of binucleated cells with MNi and NBUDs observed in cultures treated with BPs. Nucleoplasmic bridges were not observed. Tables 1 and 2 show the results of the frequencies of MNi and NBUDs in human peripheral lymphocytes cultured with different concentrations of BPs. Our results showed that BPA and BPF significantly increased the MNi frequency from a concentration of 0.025 μg/ml to the highest concentration of 0.1 μg/ml (Table 1), whereas BPS showed genotoxic properties from a concentration of 0.05 μg/ml (Table 1). Conversely, BPA induced a significant increase in NBUDs only at the highest concentration of 0.1 μg/ml, whereas BPF and BPS showed no genotoxic properties with respect to NBUDs (Table 2). MMC was found to significantly increase the formation of MNi and NBUDs compared to DMSO and negative control (Tables 1 and 2). A significant decrease in CBPI was observed only in cultures treated with MMC, 0.1 μg/ml of both BPA and BPS (Table 1), while the other BPs concentrations did not show cytotoxicity. Finally, a significant correlation between the frequency of MNi and NBUDs and the different concentrations of BPs was observed (Table 3), indicating an increase in genomic damage in a concentration‐dependent manner.

**TABLE 1:** Induction of micronuclei by bisphenol A, bisphenol F, and bisphenol S in human lymphocytes *in vitro*

Test substance	Treatment period dose (h) (μg/ml)		Cells scored	MNs	Ab.C	MN/cell ± S.D. (‰)	Ab.C/cell ± S.D. (‰)	CBPI ± S.D.
NC	–	–	4000	8	8	2.000 ± 0.816	2.000 ± 0.816	1.713 ± 0.007
0.1% DMSO	48	–	4000	15	15	3.750 ± 1.258	3.750 ± 1.258	1.649 ± 0.0046
MMC	48	0.100	4000	82	76	20.500 ± 2.648**	19.000 ± 1.414**	1.368 ± 0.034^&^
BPA	48	0.100	4000	48	48	12.000 ± 1.633**	12.000 ± 1.633**	1.542 ± 0.023^&^
	48	0.050	4000	40	40	10.000 ± 1.826*	10.000 ± 1.826*	1.620 ± 0.028
	48	0.025	4000	32	32	8.000 ± 1.826*	8.000 ± 1.826*	1.639 ± 0.010
	48	0.0125	4000	21	21	5.250 ± 0.563	5.250 ± 0.563	1.651 ± 0.016
BPF	48	0.100	4000	37	37	9.250 ± 1.258***	9.250 ± 1.258***	1.555 ± 0.079
	48	0.050	4000	32	32	8.000 ± 1.826**	8.000 ± 1.826**	1.631 ± 0.017
	48	0.025	4000	23	23	5.750 ± 0.957*	5.750 ± 0.957*	1.655 ± 0.025
	48	0.0125	4000	17	17	4.250 ± 0.563	4.250 ± 0.563	1.670 ± 0.025
BPS	48	0.100	4000	33	33	8.250 ± 1.258***	8.250 ± 1.258***	1.574 ± 0.027^&^
	48	0.050	4000	30	30	7.500 ± 1.291**	7.500 ± 1.291**	1.622 ± 0.019
	48	0.025	4000	20	20	5.000 ± 1.826	5.000 ± 1.826	1.615 ± 0.053
	48	0.0125	4000	18	18	4.500 ± 0.577	4.500 ± 0.577	1.697 ± 0.114

*Note*: All significant differences are reported with respect to DMSO (Mann–Whitney test).

Abbreviations: Ab.C, aberrant cells (cells with 1 or more MNs); BNC, binucleated cell; BPA, bisphenol A; BPF, bisphenol F; BPS, bisphenol S; CBPI, cytokinesis‐block proliferation index; MMC, mitomycin‐C; MN, micronuclei; NC, negative control; S.D., standard deviation.

& *p* ≤ .021.

* *p* ≤ .037.

** *p ≤* .020.

*** *p ≤* .018.

**TABLE 2:** Induction of NBUDs by BPs in human lymphocytes *in vitro*

Test substance	Treatment period dose (h) (μg/ml)		Cells	NBUDs	NBUDs/cells ± S.D. (‰)
NC	–	–	4000	2	0.500 ± 0.577
0.1% DMSO	48	–	4000	3	0.750 ± 0.500
MMC	48	0.100	4000	20	5.000 ± 1.414**
BPA	48	0.100	4000	10	2.000 ± 0.820*
	48	0.050	4000	5	1.250 ± 0.500
	48	0.025	4000	4	0.750 ± 0.500
	48	0.0125	4000	3	0.750 ± 0.500
BPF	48	0.100	4000	8	1.750 ± 0.957
	48	0.050	4000	4	1.000 ± 0.817
	48	0.025	4000	3	0.500 ± 0.577
	48	0.0125	4000	2	0.500 ± 0.577
BPS	48	0.100	4000	4	1.500 ± 0.577
	48	0.050	4000	3	1.000 ± 0.816
	48	0.025	4000	3	0.750 ± 0.500
	48	0.0125	4000	3	0.750 ± 0.500

*Note*: All significant differences are reported with respect to DMSO (Mann–Whitney test).

Abbreviations: BPA, bisphenol A; BPF, bisphenol F; BPS, bisphenol S; MMC, mitomycin‐C; NBUD, nuclear bud; NC, negative control; S.D., standard deviation.

* *p* = .044.

** *p* = .017.

**TABLE 3:** Multiple regression analysis to assess the relationship between BPs concentrations, genomic damage level, and HERVs/*env* gene expression

Biomarkers	β‐co	*p* Value	95% CI (lower)–(upper)
*BPA*			
MNi	0.867	<0.001*	(1.566)–(3.109)
Cells with MNs	0.867	<0.001*	(1.566)–(3.109)
NBUDs	0.654	0.006*	(0.143)–(0.707)
CBPI	−0.817	<0.001*	(−44.539) to (−18.863)
HERV‐H/env	0.913	0.030*	(0.1626)–(0.9944)
HERV‐R/env	0.8544	0.065	(−0.1133) to (0.9902)
*BPF*			
MNi	0.850	<0.001*	(1.111)–(2.339)
Cells with MNs	0.850	<0.001*	(1.111)–(2.339)
NBUDs	0.575	0.020*	(0.078) to (−0.772)
CBPI	−0.707	<0.001*	(−58.012) to (−15.738)
HERV‐H/env	0.601	0.283	(−0.598) to (0.969)
HERV‐R/env	−0.603	0.281	(−0.969) to (0.596)
*BPS*			
MNi	0.834	<0.001*	(0.884)–(1.966)
Cells with MNs	0.785	<0.001*	(0.075)–(1.998)
NBUDs	0.456	0.076	(0.029)–(0.529)
CBPI	−0.564	0.023*	(−66.404) to (−5.796)
HERV‐H/env	−0.480	0.413	(−0.957) to (0.697)
HERV‐R/env	−0.584	0.300	(0.967)–(0.614)

*Note*: The significant *p* values were highlighted with *.

Abbreviations: CBPI, cytokinesis‐block proliferation index; CI, confidence interval; HERV‐H/*env*, human endogenous retrovirus H *env* gene; HERV‐R/*env*, human endogenous retrovirus R env; MNi, micronuclei; NBUD, nuclear bud; β‐co, β‐coefficient.

### 3.2 Expression of HERV‐H/*env* and HERV‐R/*env* gene in PBMCs

**FIGURE 2:**
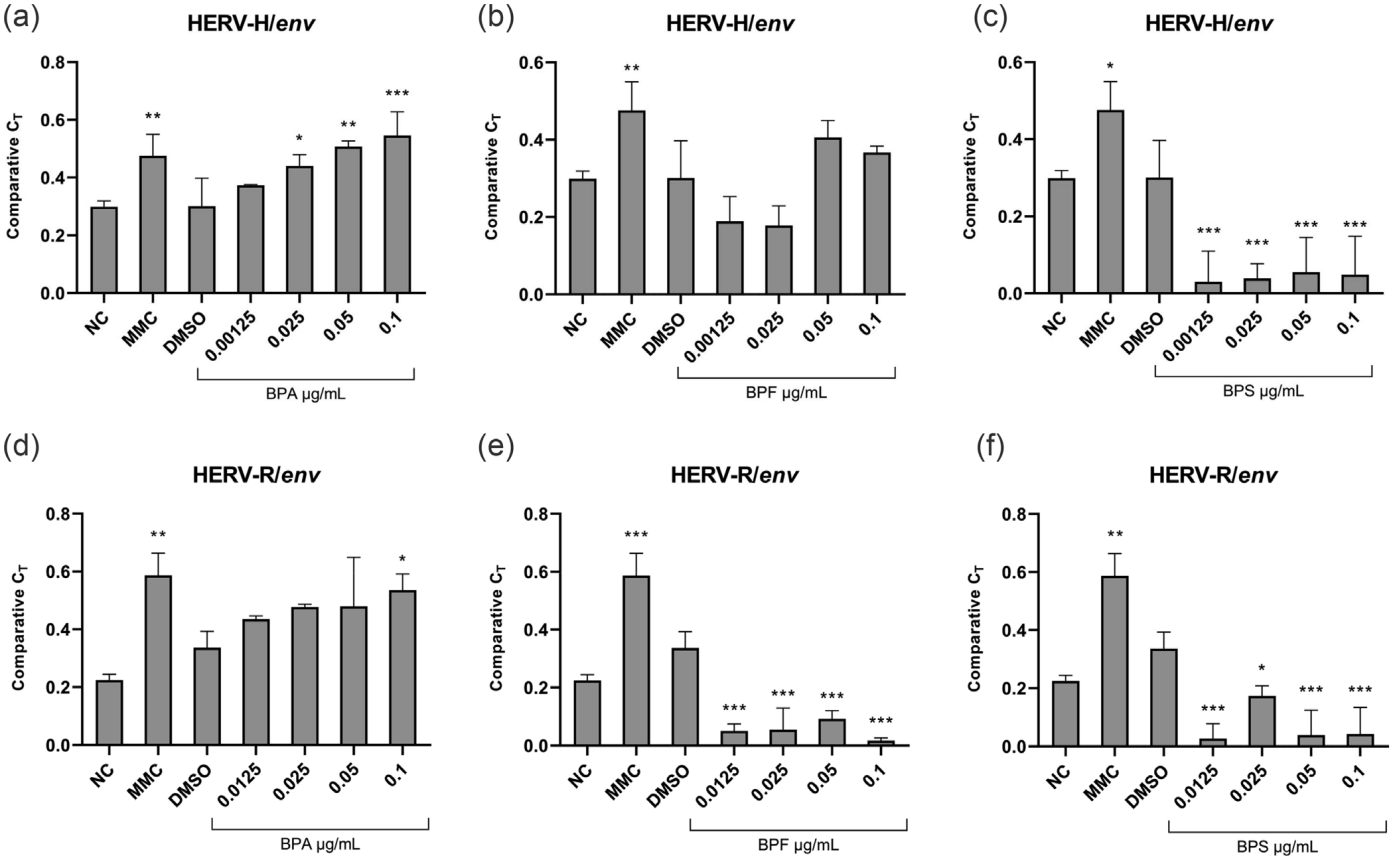
Human endogenous retrovirus H/*env* and human endogenous retrovirus R/*env* transcriptional levels in PBMCs of blood culture exposed to bisphenol A (BPA) (a–d), bisphenol (BPF) (b–e), and bisphenol S (BPS) (c–f). Negative control (NC) and positive control Mitomycin‐C (MMC). Relative *env* gene expression levels were analyzed by real‐time PCR and represented by 2^–∆∆Ct^ on a logarithmic scale. All significant differences are reported with respect to DMSO by one‐way ANOVA test: *p ≤ .043; **p ≤ .009; ***p ≤ .001.

The HERV‐H/*env* and HERV‐R/*env* genes were always transcriptionally active throughout the study sample. The primer's amplification efficiency was 110% for HERV‐H/*env* and 98% for HERV‐R/*env*. Furthermore, melting curve analysis confirmed the specificity of the amplicon without primer‐dimers and non‐specific products. NC cultures were not significantly different from those treated with DMSO, indicating that the expression of HERV‐H/*env* and HERV‐R/*env* was not significantly altered by the 0.1% DMSO concentration. Otherwise, blood cultures treated with the mutagen MMC showed a significant increase in HERV‐H/*env* and HERV‐R/*env* expression compared to DMSO solvent cultures (*p* ≤ 0.04; Figure 2).

### 3.3 Effect of BPA on HERV‐H/*env* and HERV‐R/*env* expression

As with the MNi assay, our results showed that BPA significantly increased the HERV‐H/*env* expression at concentrations equal to 0.025, 0.05, and 0.1 μg/ml (*p* ≤ .04) compared to DMSO solvent–control cultures (Figure 2a). Similarly, concerning HERV‐R/*env* expression, BPA exposure significantly increased expression only at 0.1 μg/ml (*p* = .02) than DMSO (Figure 2d). A significant correlation was found only between the expression of *env* gene in HERV‐H and the different BPA concentrations (Table 3), indicating a concentration‐dependent increase in the expression of the *env* gene. In addition, a significant correlation was found between the expression of HERV‐H/*env* and HERV‐R/*env* (*r* = .94; *p* = .01) (data not shown).

### 3.4 Effect of BPF on HERV‐H/*env* and HERV‐R/*env* expression

Compared with BPA, BPF exposure showed a differential effect on the expression of HERV‐H/*env* and HERV‐R/*env*. There was no significant difference in HERV‐H/*env* gene expression levels between exposure to different BPF doses and DMSO solvent cultures (Figure 2b). In contrast, the same exposure factors were found to dramatically reduce HERV‐R/*env* expression at all concentrations tested (0.0125, 0.025, 0.05, and 0.1 μg/ml) compared to DMSO solvent control cultures (*p* ≤ .001; Figure 2e). In addition, multiple regression analysis revealed no significant correlation between the expression of the *env* gene in either HERV and the different BPF concentrations (Table 3). Similarly, the correlation between the *env* expression of HERV‐H and HERV‐R did not yield a significant value (*r* = .10, *p* = .86; data not shown).

### 3.5 Effect of BPS on HERV‐H/*env* and HERV‐R/*env* expression

Exposure to BPS in blood cultures showed an opposite trend with respect to BPA effects. For both HERV‐H/*env* and HERV‐R/*env*, expression levels were significantly lower than DMSO solvent cultures at all BPS tested concentrations (0.0125, 0.025, 0.05, and 0.1 μg/ml; *p* ≤ .04) (Figure 2c–f). No significant correlation was found between the expression of the *env* gene in both HERVs and the different BPS concentrations (Table 3). In contrast to what was observed for BPF, the correlation between *env* expression in HERV‐H and HERV‐R yielded a significant value (*r* = .88, *p* = .047; data not shown).

### 3.6 Correlation between HERVs/*env* expression and genomic damage

**FIGURE 3:**
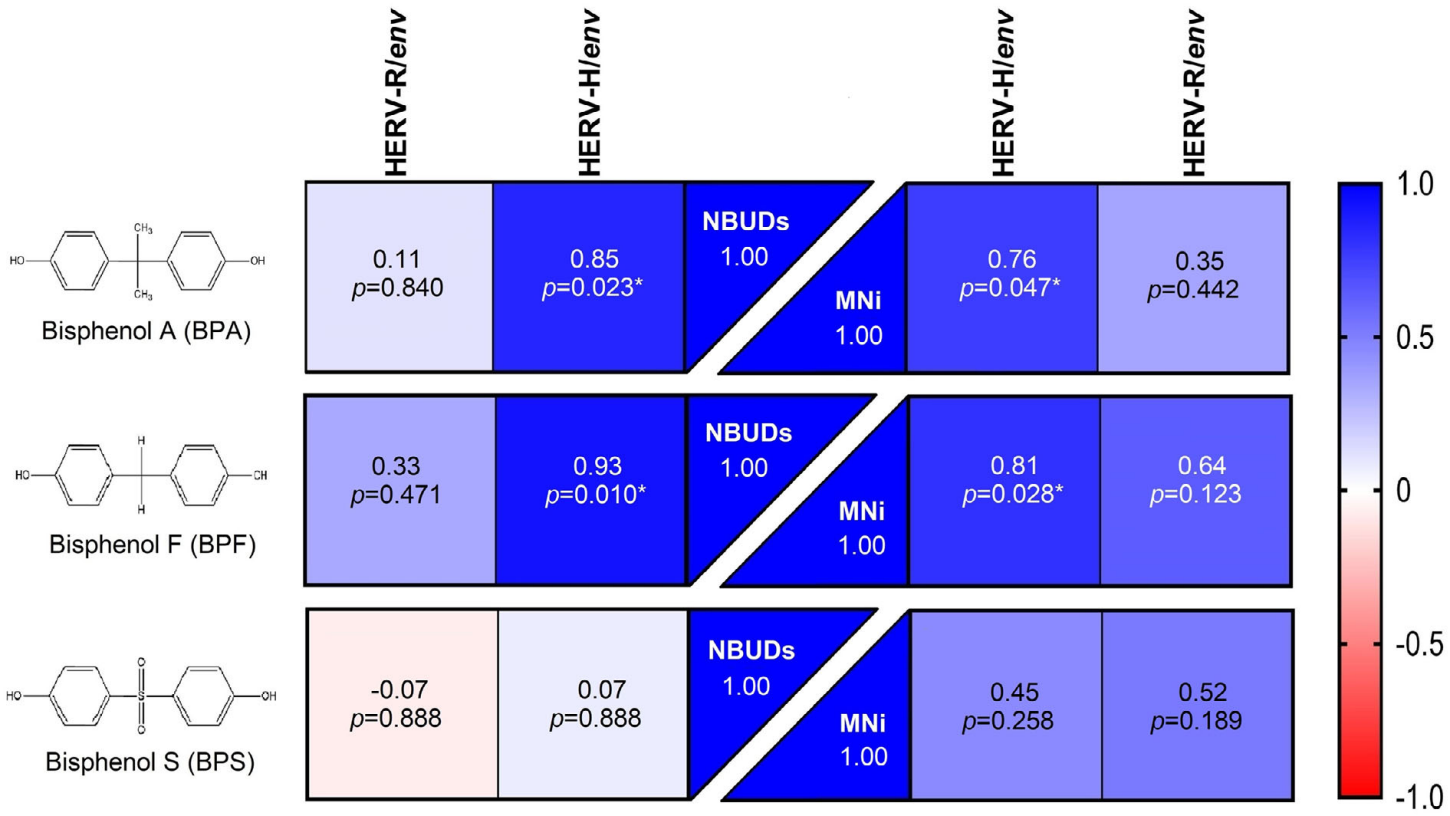
Correlation analysis between human endogenous retrovirus H/*env* and R/*env* transcriptional levels and micronuclei (MNi) and nuclear buds (NBUDs) formation in PBMCs. Parametric Pearson's matrix computes the correlation between HERVs/*env* expression level and MNi formation. Nonparametric Spearman's matrix computes the correlation between HERVs/*env* expression level and NBUDs formation. For each box at the top the *r* correlation score and at the bottom the *p* values are reported.

Different from what was observed for BPS, correlation analysis revealed significantly higher *r* values between HERV‐H/*env* expression levels and the formation of MNi and NBUDs after treatment with BPA and BPF. Vice versa, no significant correlation was observed for HERV‐R/*env* expression levels with MNi and NBUD formation for all BPs treatments (Figure 3). Of note, although not statistically significant, is that BPS exposure showed the lowest *r* values between HERVs/*env* expressions and the number of NBUDs.

## 4 DISCUSSION

The clastogenic and aneugenic properties of BPA in human cells are well documented (Di Pietro et al., 2020; Pfeifer et al., 2015). Conversely, the literature on the genomic effects of BPF and BPS is sparse (Cabaton et al., 2009; Lee et al., 2013). Therefore, we decided to evaluate the genotoxic properties of BPA and its analogs BPF and BPS in the same experiment to compare the observed induced genomic damage with the expression of HERV‐H and HERV‐R.

The results of our study confirm the genotoxic effects of BPA reported in the literature at concentrations of 0.1 and 0.05 and 0.025 μg/ml in terms of a significant increase in MNi abundance (Santovito et al., 2018). These results are also consistent with those of Tayama et al. (2008), who showed a significant increase in structural CAs, such as exchanges, breaks, and gaps, in cultured CHO‐K1 cells exposed to different BPA concentrations. A significant reduction in CBPI was observed in cultures treated with BPA at a concentration of 0.01 μg/ml (Table 1), suggesting that BPA impairs mitotic activity. Similar to BPA, BPF showed significant genotoxic effects at concentrations of 0.1, 0.05, and 0.025 μg/ml. To date, there are limited data in the literature on the genotoxicity and cytotoxicity of BPF, so an RD has not yet been established. In particular, BPF cytotoxicity was documented by Cabaton et al. (2009), who observed a significant increase in DNA breaks in HepG2 cells by the comet assay, but not in MNi frequency, indicating a beneficial effect of an efficient genomic damage repair system. However, we would like to emphasize that the authors treated HepG2 cells with BPF for 20 h, whereas in our study, blood cultures were exposed to BPF for 48 h. It seems that the additional time could be a cofactor for the increase in the micronucleus frequency. Indeed, Lee et al. (2013) reported a significant increase in the number of CAs in RAD54^−^/^−^ after 48 h of BPF incubation. Note that MNi assays detect changes in both chromosome number and structure, whereas CAs analyzes can only detect abnormalities in chromosome structure. One mechanism of action of xenobiotic compounds is the induction of modifications to centromeric DNA, resulting in the inability of damaged chromosomes to adhere to the mitotic spindle (Yüzbaşioğlu et al., 2006). As suggested by George et al. (2008), it is plausible that BPs could also induce damage to chromosome centromeres, leading to the formation of MNi without the appearance of CAs. In particular, there are several mechanisms by which genotoxic xenobiotics may cause lesions to DNA that lead to MNi formation. According to Fenech et al. (2016), chemicals may generate ROS or RNS, inhibit DNA damage response, and impact on polymerization of proteins required to form cytoskeletal structures which are essential for the mitotic process. All of these factors can lead to nuclear plasmatic bridge breaks, which in turn lead to the formation of MNi (Fenech et al., 2011; Utani et al., 2010).

Among the bisphenols analyzed in the present work, BPS seems to have less genotoxic properties, showing increased MNi abundance above a concentration of 0.05 μg/ml. This result does not seem to be reassuring, since the European Union Regulation No. 10/201 allows the use of BPS as a monomer in FCM with an SML of 0.05 mg/kg (EFSA et al., 2020). Finally, although BPS did not show the same MNi frequency as other bisphenols tested in our work, several studies reported similar non‐genomic effects of BPS, which are very similar to those of BPA (Salvesen & Walsh, 2014; Viñas & Watson, 2013). According to this, the androgen and estrogen activities of BPS and BPF are of the same order of magnitude as those of BPA, in both *in vitro* and *in vivo* studies (Rochester & Bolden, 2015).

It is known that fragmentation of dsDNA caused by acute DNA damage can lead to the accumulation of dsRNA fragments in the cytosol (Canadas et al., 2018; MacDonald et al., 2020), arising from aberrant expression of ERVs (Canadas et al., 2018; Lee et al., 2012) and SINEs (Rudin & Thompson, 2001). Although most of the human endogenous retrovirus (HERV) genes are inactive, some of them can still encode proteins. One of these is the HERV‐R/*env* gene, whose transcriptional downregulation has been reported in cases of choriocarcinoma (Rote et al., 1998). In particular, significant antibody response was observed against HERVs envelope (*env*) epitopes detected in patients with diabetes and other diseases (Manca et al., 2022; Noli et al., 2021; Simula et al., 2021). In 2009, a study revealed that the presence of these *env* viral proteins could contribute to the development of cancer cells (Balada et al., 2009).

In this study, the effects of BPs exposure on the transcriptional activity of human PBMCs were studied. We found that the three different types of BPs, namely, BPA, BPF, and BPS, exhibited different regulatory mechanisms that affect the expression of HERVs *env* genes. While the PBMC exposed to the BPA showed higher levels of transcriptional activity, the peripheral blood cells that were treated with BPS had lower levels of transcriptional activity in both of HERVs genes. The PBMCs exposed to BPF showed a significant alteration in the expression level of HERV‐R/*env* but not of HERV H/*env*. This suggests that the BPs may regulate the levels of these genes due to the activation of immune‐cytokine response and/or epigenetic factors (Hurst & Magiorkinis, 2017; Katsumata et al., 1999). (1) The effects of DNA methylation and heterochromatin‐silencing on the activation of the HERVs genes could be explained by the presence of environmental factors that can affect the DNA accessibility of these genes, such as lifestyle and pollution (Lee et al., 2012; Pathak & Feil, 2018). The increase in HERV/*env* gene expression by BPA exposure (Figure 2a–d) is consistent with the global DNA hypomethylation reported in MCF‐7 cells (Barchitta et al., 2014; Wang et al., 2018). Furthermore, DNA methylome‐wide analysis revealed that BPA has a stronger effect than BPF and BPS inducing DNA methylation aberrations, hypomethylation of oncogenes, and a decrease in apoptosis gene expression in MCF‐7 breast cells (Awada et al., 2019; Wang et al., 2018). (2) Another regulatory response may result from data published by Izumi et al. (2011) and Malaisé et al. (2020) who revealed that exposure to BPs can trigger an increase in the secretion of certain cytokines, such as IL‐1β and IFN‐γ. These factors in turn may regulate the HERV‐R/*env* transcriptional activity (Katsumata et al., 1999). (3) Finally, it is known that micronuclear envelopes are prone to rupture, whereupon cyclic GMP‐AMP synthase can recognize the enclosed dsDNA, produce cGAMP, and promote the expression of inflammatory genes via the stimulator interferon gene (STING) (MacDonald et al., 2020). The inflammatory response may be triggered by exposure to BPs and DNA rearrangement of MNi, resulting in differential expression of HERVs (Katsumata et al., 1999).

Positive and significant correlations were observed between transcriptional levels of HERV‐H/*env* and frequencies of NBUDs from BPA and BPF treatment. NBUDs were observed in cultures grown under a condition that induces gene amplification (Fenech & Crott, 2002). The nucleus eliminates excess amplified DNA by an active process that concentrates amplified DNA to the periphery of the nucleus and is eliminated via nuclear budding to form MNi during the S phase of the cell cycle (Shimizu et al., 1998). Significant increases in NBUDs formations were reported in the case of high HERV‐H/*env* expression levels, but only in BPA and BPF treatments. These results further highlight the potential of BPA and BPF to increase the gene amplification leading to NBUDs formation, whereas no significant NBUDs formation was detected in treatments with BPS, which instead showed a reduction in expression of HERVs genes.

## 5 CONCLUSIONS

The present study demonstrated that exposure to BPA and its analogs, BPS and BPF, leads to the accumulation of genomic instability in human peripheral blood cultures by increasing MNi frequencies and altering the expression of HERVs *env* genes. All samples studied clearly showed expression levels of HERVs genes, and therefore we suggest that the detection of HERVs beyond a certain gene expression range may be a cofactor for harmful conditions in the human body. Although the sample size of this study is small, strong correlations and relationships among MNi frequency, HERVs gene expression, and BPs exposure were evident. The high number of cells scored in MNi assay required resource‐intensive; therefore, these *in vitro* results set the stage for *in vivo* studies with a larger sampling. In conclusion, BPA, BPS, and BPF reported significant genotoxicity in peripheral human blood even at a concentration of RD, so these BPA analogs need further studies to better assess their long‐term impact on human health.

## AUTHOR CONTRIBUTIONS

Stefano Ruberto designed the study, data curation, formal analysis, investigation, methodology, software, writing – review and editing with important intellectual input from Alfredo Santovito and Leonardo A. Sechi. Alfredo Santovito recruited the samples and collected the data, investigation, methodology, project administration, and writing – review and editing. Elena R. Simula, Marta Noli, and Maria A. Manca investigation and methodology. Leonardo A. Sechi writing editing and project administration. All authors approved the final manuscript.

## CONFLICT OF INTEREST

The authors declare no conflicts of interest.
